# Romosozumab in osteoporosis: yesterday, today and tomorrow

**DOI:** 10.1186/s12967-023-04563-z

**Published:** 2023-09-27

**Authors:** Dong Wu, Lei Li, Zhun Wen, Guangbin Wang

**Affiliations:** 1 Department of Orthopaedics, Zhuanghe Central Hospital, Zhuanghe City, 116499 Liaoning Province China; 2 Department of Orthopeadics, Shengjing Hospital of China Medical University, No. 36 Sanhao Street, Heping District, Shenyang, 110004 Liaoning People’s Republic of China; 3 Department of Urology, Union Hospital, Tongji Medical College, Huazhong University of Science and Technology, Wuhan, 430022 China

**Keywords:** Romosozumab, Osteoporosis, Wnt/β-catenin signaling pathway, Sclerostin

## Abstract

Osteoporosis is a systemic bone disease characterized by low bone mass, microarchitectural deterioration, increased bone fragility, and fracture susceptibility. It commonly occurs in older people, especially postmenopausal women. As global ageing increases, osteoporosis has become a global burden. There are a number of medications available for the treatment of osteoporosis, categorized as anabolic and anti-resorptive. Unfortunately, there is no drugs which have dual influence on bone, while all drugs have limitations and adverse events. Some serious adverse events include jaw osteonecrosis and atypical femoral fracture. Recently, a novel medication has appeared that challenges this pattern. Romosozumab is a novel drug monoclonal antibody to sclerostin encoded by the SOST gene. It has been used in Japan since 2019 and has achieved promising results in treating osteoporosis. However, it is also accompanied by some controversy. While it promotes rapid bone growth, it may cause serious adverse events such as cardiovascular diseases. There has been scepticism about the drug since its inception. Therefore, the present review comprehensively covered romosozumab from its inception to its clinical application, from animal studies to human studies, and from safety to cost. We hope to provide a better understanding of romosozumab for its clinical application.

## Background

Osteoporosis (OP) was a systemic bone disorder characterized by low bone mass, increased bone fragility, and fracture susceptibility defined in a consensus development conference in 1993 [1]. It has been a global burden for the elderly. Over 200 million people are thought to be affected by osteoporosis worldwide [2]. According to the International Osteoporosis Foundation, one in every three women over 50 years and one in every five men may have an osteoporotic fracture during their lifetimes [3]. Bone mass density (BMD) is recommended to diagnose OP. OP and osteopenia were defined as having a T score of BMD less than − 2.5 and between − 1 and − 2.5, respectively [1, 4]. Fracture Risk Algorithm (FRAX) scoring is commonly recommended to assess the risk of fractures [5, 6]. Bone turnover markers (BTMs) assess bone remodeling [7, 8]. The majority of bone osteoid is composed of type I collagen. Therefore, BTMs are associated with type I collagen activity. Type I collagen degradation (CTX‑I and NTX‑I) and synthesis (PICP and PINP) are widely used as bone resorption markers and formation markers, respectively [9]. The objectives of OP treatment are to increase bone mass and prevent fractures [10]. The drugs used to treat OP are classified as anabolic and anti-resorptive drugs [11, 12]. The anti-resorptive drugs include bisphosphonates, raloxifene, tibolone, and denosumab. The anabolic drugs include teriparatide (parathyroid (PTH) hormone analogue) and abaloparatide (human PTH hormone-related peptide analogue) [10]. Both anabolic and anti-resorptive drugs have their limitations and adverse effects [4]. Bisphosphonates, the first line treatment for OP, have an issue of drug holidays and atypical femoral fractures [13]. Raloxifene therapy increases the risk of venous thrombosis [14]. Tibolone users had twice the risk of stroke as controls [15]. Denosumab is an anti-resorptive drug, and its anti-resorptive effect rebounds rapidly after discontinuation [16]. It is generally accepted that OP treatment takes a long time. However, the use of anabolic drugs is restricted for up to 2 years due to serious adverse events [17].

Sclerostin is a classical inhibitor of Wnt/β-catenin signaling, which is involved with many diseases, such as cancer, eye disease, and bone diseases [18–20]. When Wnt/β-catenin signaling is activated, bone mass can significantly increase. Conversely, when Wnt/β-catenin signaling is inhibited, bone mass decreases [21]. Romozumab is a cutting-edge monoclonal antibody against sclerostin that promotes bone formation and inhibites bone resorption [22]. It can rapidly increase bone mass in a short period of time, preventing fractures [23, 24]. This has never happened before. However, a lot of controversy has accompanied romosozumab [25, 26].

In the present paper, we reviewed the mechanism of romosozumab and then thoroughly evaluated the history, assessed the situation, and discussed the potential applications of romosozumab in the future.

### Sclerostin and Wnt/β-catenin signaling

The sclerostin/SOST gene first appeared due to two rare bone overgrowth diseases, sclerosteosis and van Buchem disease, mapped to chromosome 17q12-q21 [27, 28]. Loss of SOST gene function, which encodes sclerostin, was first reported in May 2001 [29]. Then, SOST was verified that it bind with low-density lipoprotein-related receptors 5 and 6 (LRP5/6), thereby suppressing Wnt/β-catenin by preventing the complex of wnt-frizzled-LRP formation [30–32]. When the Wnt ligand is not present, Axin, adenomatous polyposis coli (APC), glycogen synthesis kinase 3 (GSK3), and casein kinase 1 (CK-1) together bind to the β-catenin in the cytoplasm to form the destruction complex, which promotes β-catenin phosphorylation to regulate its stability [33, 34]. Axin is a scaffolding protein in the complex and phosphorylated LRP5/6 [35]. APC and Axin together promote β-catenin destabilization [36]. CK-1 phosphorylates β-catenin at Ser45, whereas GSK3 phosphorylates β-catenin at Ser37, Thr41, and Ser33 residues. The phosphorylated β-catenin is then recognized by E3-ubiquitin ligase, and β-catenin is ubiquitinated [37]. The above process maintains low levels of β-catenin expression in the cytoplasm. Axin protein is recruited to LRP5/6 after Wnt binds frizzled (FZD) and LRP5/6, and the Wnt/ signaling is activated. The GSK3β-mediated β-catenin phosphorylation is inhibited. Therefore, the destruction complex is relocalized to the cytoplasmic membrane, and β-catenin is released [37, 38]. Concurrently, the intracellular regions of LRP5/6 are phosphorylated by CK-1 and GSK3 to promote β-catenin stabilization and prevent its ubiquitination [39, 40].

**Fig. 1:**
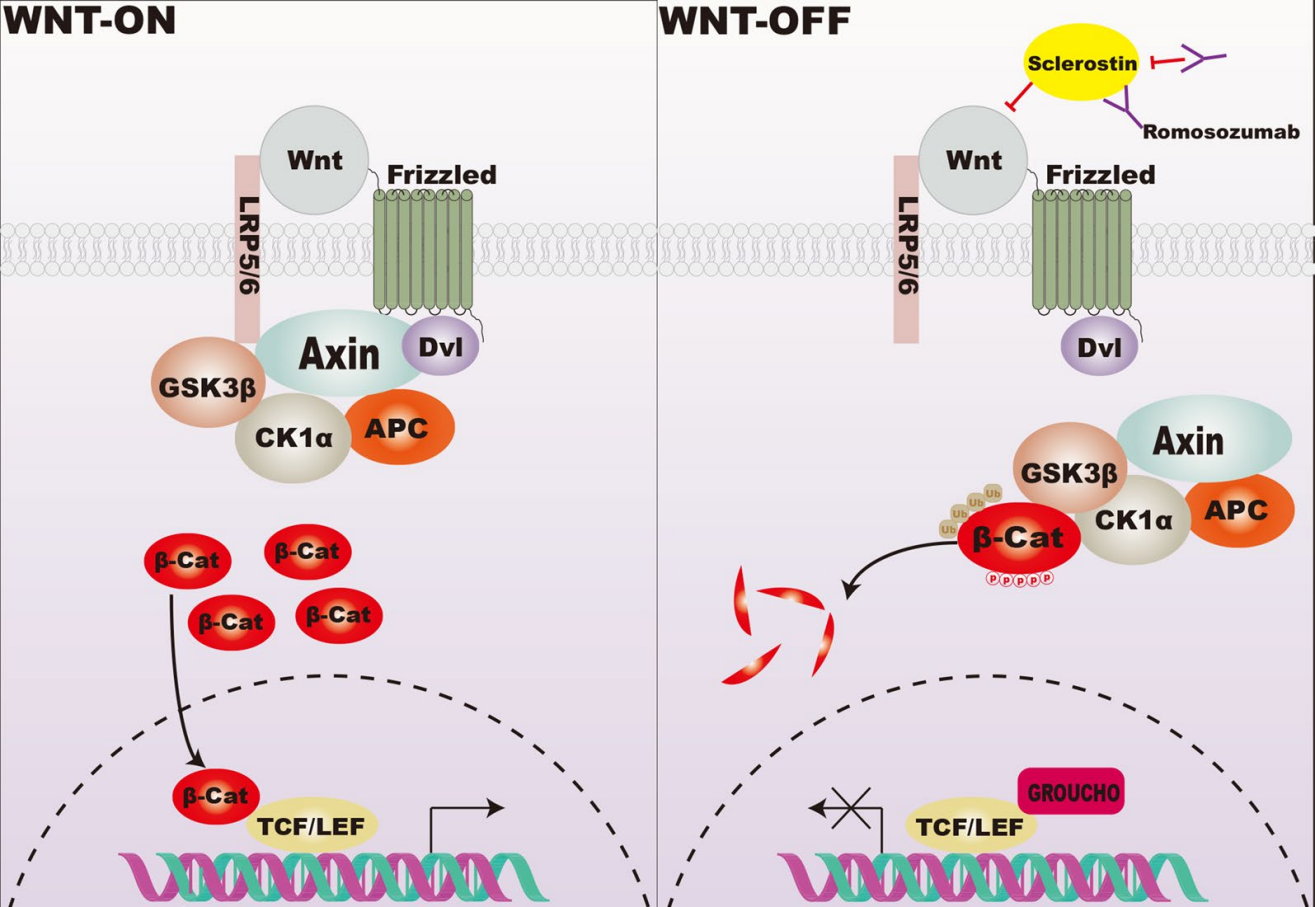
The role of sclerostin in Wnt/β-catenin signaling

Finally, the destruction complex has reached saturation, with β-catenin accumulating in the cytoplasm [37, 38, 41]. In the nucleus, β-catenin cannot bind DNA and must interact with DNA-binding proteins from the TCF/LEF family of transcription factors [42, 43]. While no signals enter the nucleus, transcription repressors of the Groucho family bind to TCF/LEF [44]. In summary, sclerostin can inhibit Wnt/β-catenin signaling, which controls the progression of osteogenic differentiation [38]. Sclerostin can thus influence bone formation by inhibiting Wnt/β-catenin signaling (Fig. 1).

### Sclerostin functions in bone

**Fig. 2:**
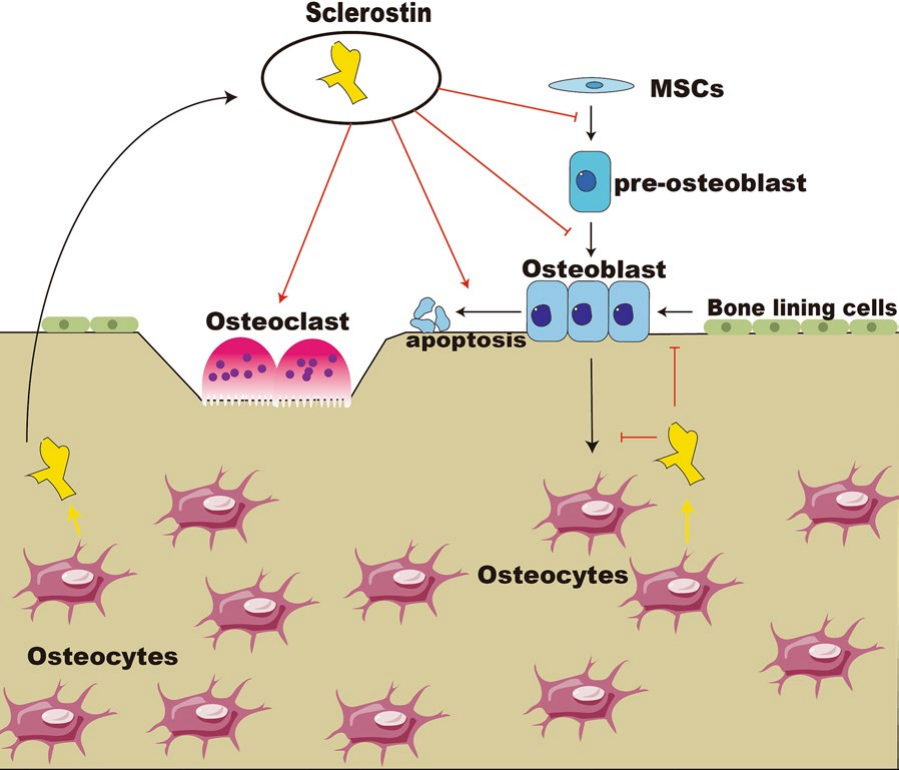
The role of sclerostin in bone

Bone remodeling and modeling are in charge of dynamic balance of bone. The role of bone modeling is to shape bone and increase bone mass. Conversely, the function of remodeling is to renew bone and maintain or slightly decrease bone mass [45, 46]. The architecture and composition of a person's skeleton are constantly remodeled to maintain its integrity throughout its lifespan [45]. During this process, bone resorption by osteoclasts and production by osteoblasts are strongly coupled within a basic multicellular unit (BMU), and bone resorption always occurs before bone formation [46]. Skeletal cells that respond to bone remodeling are classified into osteoblast, osteoclast, and osteocytes [47]. Osteocytes are the most abundant and can secrete sclerostin. Large multinucleated cells known as osteoclasts, which are derived from hematopoietic stem cells (HSCs), are in charge of bone resorption [48]. Osteoblast originates from mesenchymal stell cells (MSCs) [49]. Osteoblast undergoes apoptosis, gets embedded in mineralized bone matrix, or remain dormant on the bone surface as bone lining cells in cortical bone [50]. Interaction between them helps to maintain bone homeostasis [51, 52]. Previous reports revealed that osteocytes secrete sclerostin (SOST) and act in a paracrine manner to prevent osteoblast differentiation and bone production [53]. Then, sclerostin promotes human osteoblast apoptosis by interacting with bone morphogenetic protein (BMPs) to inhibit bone formation [54]. In a previous study, MSCs extracted from the mice treated with sclerostin antibody demonstrated a greater capacity for osteogenic differentiation [55]. Ominski et al*.* reported that sclerostin neutralization promoted osteoprogenitor cell proliferation and recruitment to active surfaces, increasing osteoblast quantity and bone mass [56, 57]. Osteocytes can be formed in the late stage of osteoblast development. Sclerostin was demonstrated to inhibit this progression in vitro [58]. Simultaneously, sclerostin antibody (Scl-ab) was proven to promote the conversion of lining cells into active osteoblasts, thereby stimulating bone formation [50, 55]. While osteocytes have the ability to resorb localized mineral [59]. The membrane-associated cytokine receptor activator of nuclear factor-kappa B ligand (RANKL) primarily mediates osteoclastogenesis [60]. Osteoprotegerin (OPG), produced by osteoblast, binds to the RANKL receptor and inhibits osteoclastogenesis [60]. The increase of RANKL in daßcat(Ot) mice is dependent on SOST/sclerostin; Sclerostin inhibits Wnt signalling resulting in inhibition of osteoblastogenesis, which reduces OPG expression and raises the RANKL/OPG ratio, causing bone resorption [21, 61] (Fig. 2).

### Romosozumab in osteoporosis

#### Pre-clinical study

Before the human study, SOST KO mice had showed significantly increased BMD, bone volume (BV), bone production, and bone strength (BS) [62]. This evidence suggests that anti-sclerostin therapy could be used to regulate bone mass. Following that, a pre-clinical study demonstrated that antibody against sclerostin completely reversed the bone loss in ovariectomized rats which is considered as good models for animal experiments and increased bone mass and BS to higher levels [63, 64]. The finding suggests a promising therapeutic approach to treating OP. Subsequently, in a female cynomolgus monkey study, monkey receiving anti-sclerostin achieved the desired results of increased BMD and BS [65]. All these pre-clinical trials suggest that sclerostin monoclonal antibody is destined to become an effective novel OP treatment option.

#### Phase 1 study

The first human study of anti-sclerostin (AMG785) was published in 2010 [66]. This study included 72 participants who received AMG 785 at various doses ((0.1, 0.3, 1, 3, 5, or 10 mg/kg) subcutaneously (s.c.) or (1 or 5 mg/kg) intravenously (i.v.)). AMG 785 serum concentrations peaked within the first week of s.c. treatment. In the highest s.c. and i.v. dose groups, serum concentrations of AMG 785 fell in a biphasic form with apparent beta (11–18 days) and gamma (6–7 days) half-lives. Participants who received a dose of AMG 785 had increased bone-formation markers in a dose-dependent manner and decreased bone resorption markers. Except for the 5 mg/kg group's total hip BMD on day 29, patients who received AMG 785 had higher BMDs at total hip (TH) and lumbar spine (LS) at each time point than individuals who received a placebo. On day 85, the 10 mg/kg s.c. group had the highest increase in BMD at LS (5.3%) and TH (2.8%). The majority of adverse events (AE) were considered mild. However, a 10 mg/kg s.c group subject experienced hepatitis-related serious adverse events (SAE) [66]. In the first-in-human study, AMG 785 produced a significant anabolic window by increasing bone formation while decreasing bone resorption. Simultaneously, AMG 785 significantly increased BMD and was well-tolerated. This study provided support for AMG 785 in further clinical research.

#### Phase 2 study

A phase 2 study was published in 2014 to evaluate the efficacy and safety of romosozumab. A total of 419 postmenopausal women with low BMD were enrolled in the study. Participants were given romosozumab at various doses at random (70, 140, or 210 mg s.c. every month or 140 or 210 mg s.c. every 3 months). Alendronate (oral, 70 mg weekly) or teriparatide (s.c. 20 µg daily) and s.c. placebo was set as open-label groups. At month 12, participants in all the romosozumab groups had higher BMD. Compared with the baseline, the group receiving 210 mg romosozumab every month showed the maximum increase in BMD (LS 11.3%, TH 4.1%, and femoral neck (FN) 3.7%). BMD gains were significantly higher than alendronate and teriparatide (p < 0.001). No significant BMD gain was observed at the distal third of the radius compared to alendronate and teriparatide. The P1NP level peaked at month 1 but dropped since month 2 and remained below baseline at month 12. CTX was significantly reduced in the first month, returned to baseline at month 3, and remained below baseline at month 12. The AE and SAE reported were comparable to placebo groups [67]. A total of 17 SAE occurred in the romosozumab group. In summary, romosozumab increased BMD greater than alendronate and teriparatide.

Desmond Padhi et al*.* reported another phase 2 study involving 32 women and 16 men with low bone mass. This study aimed to assess romosozumab treatment at various doses and administration intervals. Men were given 1 mg/kg Q2W or 3 mg/kg Q4W or placebo, while women received six doses of 1 or 2 mg/kg every 2 weeks (Q2W) or three doses of 2 or 3 mg/kg every 4 weeks (Q4W) for 6 weeks. P1NP levels were highest in the men 3 mg/kg Q4W group (33%, compared with baseline), and in women 1 mg/kg Q2W (22%) and 3 mg/kg Q4W group (21%). The most significant CTX decrease was observed in the 2 mg/kg Q4W in the women group (8.7%), followed by the 3 mg/kg Q4W men group (4.8%). P1NP increased sharply in the first 8 weeks of the phase 1 study and then decreased. CTX levels dropped significantly in the first 2 weeks before returning to baseline. At LS, BMD in all romozozumab was higher (Max7%). BMD also increased at TH (3%) in the 2 mg/kg Q2W and 3 mg/kg Q4W groups. Similar increases were observed at specific times and sites (femoral neck, wrist, radius, and total body). The AE and SAE were comparable. No death was reported. One patient had severe coronary artery disease; another had hematochezia and acute blood loss anemia. Two patients' AE were unrelated to the study. The study demonstrated that romosozumab increased bone formation and decreased bone resorption. Simultaneously, romosozumab was well-tolerated in multiple doses [68]

Furthermore, Japanese researchers reported a phase 2 study to assess romosozumab at different doses (70, 140, or 210 mg s.c. every month) for 12 months. A total of 252 Japanese osteoporotic patients were enrolled in the study and were randomly assigned to receive romosozumab (70 mg, 140 mg, or 210 mg) or placebo. At month 12, the BMD in the romosozumab 210 mg QM group increased significantly (p < 0.01, compared with the placebo group) at LS, TH, and FN (16.9%, 4.7%, and 3.8, compared with baseline). P1NP increased significantly in the first month and remained below baseline by month 12. In contrast, CTX levels were lowest at week 1 and remained below baseline at every study timepoints. The AEs and SAEs in patients were generally comparable across groups. There was no serious AEs associated with romosozumab therapy [69]. The findings were consistent with the previous phase 2 international study [67, 69]. The results confirmed the safety and efficacy of romosozumab in Japanese women.

All previous trials used romosozumab for a maximum of 1 year. So, can the duration of romosozumab treatment be extended further? Is there significantly more bone mass gain or AEs after the extended period? This question was answered in 2018 by a phase 2 international extension study. The first 12 months' results were reported previously [67]. Within the next 12 months, the alendronate group was switched to romosozumab 140 mg s.c., and teriparatide groups were discontinued. After 24 months, patients in different groups were randomly assigned to receive a placebo or denosumab 60 mg Q6M for 12 months. Similar to the results in month 12, patients in the romosozumab 210 mg QM groups had the highest BMD gain at month 24 (LS 15.1%, TH 5.4%, and FN 5.2%; p < 0.01). At the same time, BMD was increased in the switched group at month 24 (LS 9.0%, TH 2.6%, and FN 2.6%). Additional mean BMD increases were observed in romosozumab 210 mg QM groups at month 36 (LS 2.6%, TH 1.9%, and FN 1.4%). The bone turnover markers changed as in the previous study [67]. However, BMD increments observed in the second year were smaller than those observed in the first year, consistent with bone turnover markers results [70]. Romosozumab provided the most significant benefit in the first year. When romosozumab was discontinued, BMD returned to baseline compared with romosozumab, followed by denosumab, implying that an anti-resorptive drug followed by romosozumab is vital.

Simultaneously, the findings revealed that romosozumab was well-tolerated over 2 years. AEs and SAEs were both balanced [70]. This study provided critical data about long-term efficacy and safety for romosozumab administration for 2 years. It also provided information for other phase 3 studies.

In 2019, a second course of romosozumab was reported. It is a phase 2 dose-finding study based on previous research [67, 70]. After 36 months, all enrolled patients were given romosozumab for 12 months (months 36–48). The BMD gain, bone turnover markers changes, and safety in the second course were comparable to the first course. These findings demonstrated that the responsiveness to romososumab had completely reset in the placebo (months 24–36) groups [71]. The eligible patients were given 5 mg zoledronate at month 48, while patients who received romosozumab in the first 24 months were given no further active treatment. Compared with BMD at month 48, BMD at month 72 decreased in patients who did not receive further active treatment but maintained in patients with a dose of 5 mg zoledronate. In patients without further active treatment, P1NP decreased since month 48 but remained above the baseline at month 72. P1NP levels in patients receiving 5 mg zoledronate decreased and remained below the baseline at month 72. CTX levels were elevated and remained baseline at month 72 in patients with no further active treatment. CTX sharply reduced in the first 3 months after receiving a dose of 5 mg zoledronate and then returned and remained below baseline at month 72. The study demonstrated that a dose of zoledronate could maintain BMD for up to 2 years after romosozumab administration for a year. The 6-year trial provided an option and therapeutic strategy for patients at high risk of fractures.

#### Phase 3 study

In 2016, 7180 patients were enrolled in a study of romosozumab treating OP. The Fracture Study in Postmenopausal Women with Osteoporosis (FRAME) aimed to assess fracture risk and efficacy following romosozumab treatment. The patients were given romosozumab (210 mg) or placebo monthly for 1 year, randomly. The patients were then given denosumab Q6M for 1 year. The results showed that at month 12, patients who received romosozumab had a 73% lower risk of new vertebral fracture and a 36% lower risk of clinical fracture than those who received placebo. The patients treated with romosozumab had a 75% lower risk of new vertebral fracture than placebo at month 24. However, the two groups have no differences in clinical fracture risk. The findings of a study indicated that the BMD of romosozumab groups increased at LS, TH, and FN after 6, 12, 18, and 24 months of treatment [67]. The level of PINP increased significantly in the first month but decreased in month 9.

Β-CTX levels were lower at months 12 and 24. There are no differences in AEs between the two groups. However, two SAEs were reported that include atypical femoral fracture (AFF) and osteonecrosis of the jaw (ONJ) [72]. Although rare, there is still a need to be vigilant about the safety of romosozumab. Safety may take longer to manifest. There is no doubt that large-scale romosozumab studies with larger samples have demonstrated a beneficial effect on both bone mass growth and fracture risk reduction.

The FRAME extension study, published in 2018, aimed to evaluate the efficacy, safety and risk of fractures of 2 years of denosumab following 1-year romosozumab. The findings revealed that the risk of fracture was lower in romosozumab to denosumab groups than in the placebo to denosumab groups. The incidences were as followed: 1.0% vs 2.8% (new vertebral fracture), 4.0% vs 5.5% (clinical fracture), 3.9% vs 4.9% (non-vertebral fracture). The result of new vertebral fracture risk is significant (p < 0.001). Both groups indicate an increase in BMD. At month 36, the mean BMD percentage changes from baseline in the romosozumab to denosumab group were significantly higher than in the placebo to denosumab group (LS 18.1% vs 7.5%, TH 9.4% vs 4.2%, and FN 8.2% vs 3.4%). P1NP and CTX levels were reduced below the baseline at month 36. AEs and SAEs were balanced between the two groups [73]. In this 3-year FRAME trial, a year of romososumab administration can benefit patients with low BMD, a high risk of fractures, or a history of fractures for up to 3 years.

Bisphosphonates are widely used as first-line therapy for OP. Patients who do not respond to bisphosphonates and are at high risk of fractures commonly need to switch to bone-forming medications. Therefore, a study called STUCTURE was conducted in 2017. This study aimed to compare romosozumab with teriparatide in treating OP after bisphosphonates treatment. A total of 436 osteoporotic patients were randomly given romosozumab (210 mg, s.c. monthly) or teriparatide (20 µg, s.c. daily) for 1 year. The results revealed that patients in the romosozumab group had higher BMD (TH 3.2%; FN 3.4%; LS 4.4%; all groups p < 0.05) than those in the teriparatide group. PINP increased significantly in the romosozumab group in the first month but decreased and returned to baseline at month 12. CTX in the romosozumab group significantly reduced at 14 days and month 1, then returned to baseline at 3 months. Conversely, in teriparatide, PINP and CTX levels remained significantly higher than at baseline. The AEs were balanced between two groups [74]. In summary, although the length of the study was only 1 year, it suggested that patients who switch from bisphosphonates to romosozumab may have a special advantage.

In 2017, a phase 3 international study (ARCH) was reported to evaluate the fracture risk of romosozumab and alendronate in osteoporotic women. 4093 patients were given romosozumab (210 mg, s.c. monthly) or alendronate (70 mg, oral, weekly) for 1 year, randomly. The participants were then given alendronate (70 mg, oral, weekly) for another 1 year. The finding revealed that patients receiving romosozumab had a 48% lower risk of new vertebral fractures, a 27% lower risk of clinical fractures and a 19% lower risk of new non-vertebral fractures than those receiving alendronate alone at month 24. At LS (3.7% vs 5.0% at month 12, 15.2% vs 7.1% at month 24, and 14.9% vs 8.5% at month 36), at TH (6.2% vs 2.8% at month 12; 7.1% vs 3.4% at month 24; and 7.0% vs 3.6% at month 36), BMD gain was higher in the romosozumab followed alendronate groups than in the alendronate alone groups at every timepoints. PINP/CTX changed after romosozumab administration, as reported in the STUCTURE study at the first 12 months, and remained lower than baseline after oral alendronate [74]. PINP and CTX were observed lower than baseline at each time point in patients receiving romosozumab. Although AE was comparable between both groups and no cases of ONJ or AFF were observed during the double-blind period, more cardiovascular diseases (CVD) were reported (Romosozumab group 2.5% vs alendronate group 1.9%). Myocardial ischemia occurred in 16 patients receiving romosozumab and six receiving alendronate (0.8% vs 0.3%). These findings imply a significant advantage and challenge the widely used first-line alendronate therapy for women with a history of fracture [75].

Next, does it have the same effect on male osteoporotic patients? In 2018, a phase 3 study (BRIDGE) was conducted to assess romosozumab in men with OP. A total of 245 patients were randomly given (2:1) romosozumab 210 mg (s.c. monthly) or placebo for 12 months. The results revealed that the BMD increase in the romosozumab group was more significant than the placebo group (LS 12.1% vs 1.2%, TH 2.5% vs − 0.5, and FN 2.2% vs − 0.2%) at month 12. PINP levels peaked in the first month and then declined to baseline in the 6th month. PINP levels in the romosozumab group were lower than in the placebo group. The CTX level decreased the most in the first month and returned to baseline at month 3. Subsequently, CTX continued to decline until month 12 and remained lower than in placebo groups. The AEs were balanced between two groups. AFF or ONJ were not seen. However, eight patients receiving romosozumab and two receiving placebo experienced cardiovascular SAEs (4.9% vs 2.5%) [76]. The study demonstrated that romosozumab could effectively treat male OP patients, while the development of cardiovascular diseases by romosozumab needs to be considered.

A recent study of romosozumab treating OP was reported in 2021 by Korean researchers. A total of 76 postmenopausal patients with OP were assigned randomly to given romosozumab (210 mg, s.c. every 4 weeks) or placebo for 6 months. The patients receiving romosozumab had a significant BMD increase than patients receiving placebo group at month 6 (LS 9.5%vs − 0.1%, TH 2.9 vs 0.3%, and FN 3.0% vs 0.8%). P1NP peaked at month one and decreased to baseline at month 6. CTX expression remained lower in the romosozumab group at each time point. No reports of serious cardiovascular events, AFF, or ONJ [77]. The results obtained from Korean patients were comparable to those from the large sample studies. The study again demonstrated the role of romosozumab in bone formation. However, the population is small, the duration of romosozumab administration is short, the risk of fractures is not evaluated, and no sequence treatment results are reported.

Concerning ethnicity, Asian researchers analyzed the outcome of romosozumab for Japanese women in FRAME and East Asian patients in ARCH, and the results were comparable to the overall population [78, 79]. Another study used bone histomorphometry and microcomputed tomography to explain the role of romosozumab in bone tissues from 107 OP women in the FRAME after two and 12 months of treatment. As reported previously, the changes in bone tissues with increased bone mass and improved microarchitecture in the romosozumab group would reduce the risk of fractures [67, 80]. The clinical trials were as listed in Table1.

**Table1:** Clinical trials of romosozumab efficacy

Phase	Participants	Design	Outcome
Phase1	72 healthy men and PM women	Safety and efficacy	Maximum BMD increase LS 5.3%; TH 2.8%; tolerant
Phase2	419 PM women with low BMD	70, 140, or 210 mg s.c. monthly or 140 or 210 mg s.c. Q3M (12 months)	LS11.3%; TH 4.1%; FN (3.7%); Maximum BMD increase in the 210 mg s.c monthly
	48 participants with low bone mass	Dose and administration intervals study	Maximum BMD increase LS 7%; TH 3%;
	252 participants with OP	Dose and administration intervals study	LS 11.3%; TH 4.7%; FN 3.8%; Maximum BMD increase in the 210 mg s.c monthly
Phase3	7180 PM patients with OP (FRAME)	Fracture risk and efficacy	The new fracture risk and clinical fracture risk are lower
	436 patients with OP (STRUCTURE)	Efficacy compared with teriparatide after bisphosphonates treatment	Higher BMD (TH 3.2%; FN 3.4%; LS 4.4%)
	4093 women with OP (ARCH)	Fracture risk compared with alendronate	BMD increased at LS, TH, and FN at every timepoints; The new fracture risk and clinical fracture risk are lower
	245 men with OP (BRIDGE)	Efficacy in male patients	Higher BMD (LS 12.1%; TH 2.5%; FN 2.2%)
	76 PM women with OP	Efficacy	Higher BMD (LS 9.5%; TH 2.9%; FN 3.0%)

*PM* postmenopausal, *OP* osteoporosis, *BMD* bone marrow density, *LS* lumbar spine, *TH* total hip, *FN* femoral neck, *FRAME* The Fracture Study in Postmenopausal Women with Osteoporosis, *STRUCTURE* Open-Label Study to Evaluate the Effect of Treatment with Romosozumab or Teriparatide in Postmenopausal Women, *ARCH* Active-Controlled Fracture Study in Postmenopausal Women with Osteoporosis at High Risk, *BRIDGE* Placebo-Controlled Double-Blind Study Evaluating the Efficacy and Safety of Romosozumab in treating Men with Osteoporosis, *s.c.* subcutaneously

### Romosozumab in secondary osteoporosis

In 2021, a single-center, non-randomized, observational study of romosozumab in OP patients on maintenance hemodialysis was published. There were 96 romosozumab-treated (76 patients completed 1 year of administration, s.c. 210 mg, every 4 weeks), and 55 romosozumab-untreated patients were enrolled. The results indicated that after 1-year romosozumab administration, BMD was increased at LS at month 6 (baseline 0.750 ± 0.144 g/cm^2^, 0.806 ± 0.126 g/cm^2^ (p < 0.05)) and month 12 (baseline 0.750 ± 0.144 g/cm^2^, 0.849 ± 0.128 g/cm^2^ (p < 0.0001)), and at FN at month 6 (baseline 0.439 ± 0.073 g/cm^2^, 0.451 ± 0.069 g/cm^2^) and month 12 (baseline 0.439 ± 0.073 g/cm^2^, 0.470 ± 0.070 g/cm^2^ (p = 0.0091)). BMD was not increased in romosozumab-untreated patients at LS (baseline 0.789 ± 0.135 g/cm^2^, 0.795 ± 0.139 g/cm^2^ at month 12) and at FN (baseline 0.512 ± 0.105 g/cm^2^, 0.505 ± 0.109 g/cm^2^ at month 12). Bone alkaline phosphatase (BAP) increased sharply at month 1 and decreased at month 6 but remained above baseline. P1NP increased in the first 3 months but decreased in the sixth. The changes in serum TRACP-5b level were not statistically significant. There were three patients with proximal femoral fractures in romosozumab-treated groups and three patients with spinal fractures in romosozumab-untreated groups. The Cardiovascular disease occurred in five out of 96 patients in the romosozumab-treated groups and six out of 55 in the romosozumab-untreated groups. The evidence suggests that romosozumab can improve BMD in patients with OP who are on maintenance hemodialysis. Simultaneously, romosozumab would not increase the risk of fractures and cardiovascular diseases [81]. This implies that romosozumab can be used to treat other types of OP. However, it should be noted that this was not an RCT study, the population was small, and the study duration was short.

Miller et al*.* reached the same conclusion after analyzing postmenopausal women with OP and mild-to-moderate chronic kidney disease (CKD) in ARCH and FRAME. The study compared the efficacy of romosozumab to placebo or alendronate in OP patients with CKD. At month 12, the BMD in patients receiving romosozumab was significantly higher than in patients receiving placebo (FRAME) or alendronate (ARCH). Regardless of kidney function, patients receiving romosozumab have a lower fracture risk than patients receiving a placebo, which is statistically significant. Patients with normal kidney function and moderate CKD have a lower risk of fractures in ARCH. The AE and SAE were balanced between romosozumab and placebo or alendronate groups. Kidney function was stable in FRAME and ARCH during the 12 months [22].

### Romosozumab in bone fractures healing

A total of 402 patients were enrolled in phase 2 double-blinded, randomized, dose-finding study about romosozumab in fresh unilateral tibial diaphyseal fractures. Romosozumab was given to 299 patients, while 103 received a placebo. The outcomes showed that romosozumab did not accelerate bone fracture healing regardless of the doses [82]. Concurrently, another phase 2 dose-finding study of romosozumab in hip fractures described similar results. Any doses of romosozumab did not accelerate the hip fractures in clinical or radiographic outcomes [83]. It is unclear whether romosozumab can help patients with delayed healing or nonunion.

### Comparison with Denosumab

Denosumab, another classical anti-resorptive monoclonal antibody, was targeting the RANKL signaling pathway. However, the sequence of romosozumab to denosumab treatment is not specific despite previous studies on 1-year romosozumab administration followed by 2-year denosumab administration and no directly comparable study between the two drugs. A study comparing romosozumab and denosumab for BMD and disease activity over 6 months in rheumatoid arthritis (RA) patients with severe OP was published in 2021. A total of 50 patients were enrolled and randomly assigned to romosozumab or denosumab groups. The erythrocyte sedimentation rate (ESR), BMD, and disease activity score in 28 joints (DAS28) were assessed. The BMDs for TH and FN in both groups did not differ significantly at any time point. When compared to baseline, the ΔBMD in the romosozumab-treated patients increased (LS 4.9% ± 4.7% (p < 0.001), TH 1.0% ± 3.7% (p = 0.294) and FN 1.1% ± 4.6% (p = 0.335)) at month 3, and (LS 5.2% ± 7.3% (p = 0.013), TH 1.9% ± 3.2% (p = 0.038) and FN1.8% ± 3.6% (p = 0.226)) at month 6, respectively. In comparison to romosozumab-treated patients, ΔBMD in the denosumab-treated patients increased (LS 2.3% ± 4.3% (p = 0.066), TH 1.4% ± 2.6% (p = 0.072) and FN 0.7% ± 4.3% (p = 0.728)) at month 3, and (LS 3.2% ± 3.6% (p = 0.014), TH 1.8% ± 2.3% (p = 0.007) and FN 2.3% ± 3.3% (p = 0.046)) at month 6. The patients who were given romosozumab had a significantly higher BMD for LS (p = 0.044) at month 3 but not significantly at other time points. P1NP increased at month 3 (116.5% ± 229.1%) and month 6 (106.9% ± 245.8%), while TRACP-5b (bone resorptive markers) decreased at month 3 (− 30.7% ± 45.2%) and month 6 (− 21.5% ± 56.1%) in the romosozumab group. P1NP increased by 47.4% ± 26.9% and 43.4% ± 29.7% in the denosumab group, while TRACP-5b decreased by − 63.6% ± 25.8% and − 49.4% ± 27.8% than in the romosozumab group. Romosozumab has a higher bone formation capacity, whereas denosumab has a higher capacity for bone anti-resorption. The ΔDAS 28-ESR levels in the romosozumab/denosumab groups were 0.25 ± 0.58/0.07 ± 0.53 at month 3 and 0.17 ± 0.58/0.00 ± 0.78 at month 6. No significant DDAS 28-ESR difference was observed between both groups [84]. The results demonstrated that romosozumab treatment increases LS BMD more effectively than denosumab treatment while not affecting RA disease activity in RA patients with OP.

A 12-month retrospective study of the efficacy of romosozumab and denosumab in 265 postmenopausal OP patients was reported. Romosozumab was given to 131 patients, while 134 patients received denosumab. In both groups, 69 patients were studied. BMD increased at LS 7.4% ± 1.7 (p < 0.001) and 12.5% ± 2.4 (p < 0.001) at month 6 and 12, respectively, in the romosozumab group, whereas BMD increased 6.0% ± 4.1 (p < 0.001) and 7.2% ± 4.3 (p < 0.001) in the denosumab group, at month 6 and 12. Both groups were compared to the baseline. The percentage of BMD changes in the romosuzmab group was higher at 6 months (p < 0.01) and 12 (p < 0.001) than in the denosumab group. The percentages of BMD changes in the denosumab group were 2.4% and 3.6% (TH) and 2.0% and 2.6% (FN) at months 6 and 12, respectively. The percentages of BMD changes in the romosozumab group were 3.4% and 6.0% (TH) and 3.0% and 5.5% (FN) at months 6 and 12, respectively. The BMD increase data were significant (p < 0.01) compared to the baseline. At month 12, there were significant differences observed between two groups (TH p < 0.05 and FN p < 0.01). P1NP levels in the denosumab group decreased significantly at months 6 and 12 (63.1%, p < 0.001, and 68.2%, p < 0.001, respectively). P1NP was significantly higher in the romosozumab group at 6 months (5.9%; p < 0.01) and remained below baseline at 12 months (− 5.6%; p = 0.705). The difference was significant at 6 months (p < 0.001) and 12 (p < 0.001). Patients receiving denosumab had significantly lower serum TRACP-5b concentrations at month 6 (− 56.0%; p < 0.001) and 12 months (− 60.5%; p < 0.001). Patients receiving romosozumab had lower TRACP-5b levels at month 6 (− 32.1%; p < 0.001) and month 12 (− 42.9%; p < 0.001). A significant difference was observed between the groups at both time points (p < 0.001). The fracture risk was comparable between the two groups. A secondary, post-hoc analysis [85] based on FRAME [67] and FREEDOM and FREEDOM extension [86] is reported. This study highlights that romosozumab, followed by denosumab (FRAME), increased BMD more quickly and reduced fracture risks more significantly than denosumab alone (FREEDOM and FREEDOM extension).

All of the studies revealed that romosozumab could increase BMD more quickly and effectively than denosumab, implying that romososumab has distinct advantages and benefits for women at high fracture risk compared to denosumab. This disparity could be due to the different mechanisms of the two drugs. Romosozumab promotes bone formation while also inhibiting bone resorption. In contrast, denosumab only suppresses bone resorption. Regrettably, no RCT study with a large sample has been reported, and the follow-up period is relatively short.

### Cost-effectiveness

Romosuzumab is a relatively expensive novel drug compared with alendronate. Is there a cost advantage to using romosozumab to treat OP? A study determined the relative cost-effectiveness of romosozumab in Swedish osteoporotic patients. Every patient was assumed to have a recent major osteoporotic fracture. A Markov structure simulates fractures, expenses, and quality-adjusted life years (QALYs). Patients were given alendronate for up to 48 months after a 1-year romosozumab administration. The patients were given alendronate for 60 months as control group. Cost-effectiveness was calculated using the incremental cost-effectiveness ratio (ICER) and QALYs as efficacy indicators. A patient receiving romosozumab and alendronate is expected to accrue 8.547 QALYs at €60,396, and a patient in the alendronate alone group is expected to accrue 8.458 QALYs at the cost of €57,394.

Regarding incremental QALYs, romosozumab groups was associated with 0.089 more QALYs at €3002 than alendronate alone, resulting in an ICER of €33,732. While assuming the efficacy of alendronate in treating hip fractures, the sequence of romosozumab to alendronate was shown to be more affordable (lower cost and higher QALYs), as supported by Deterministic sensitivity analysis [87]. The findings revealed that the romosozumab-to-alendronate strategy might cost less in postmenopausal women with OP and a recent fracture. Japanese researchers published another study on cost-effectiveness. Romosozumab and teriparatide were used to treat severe postmenopausal OP women who had previously received bisphosphonates. The two sequential strategies were romosozumab to alendronate (1 + 4 years), and teriparatide to alendronate (2 + 3 years). The Markov model was adopted to analyze the data. The romosozumab to alendronate strategy saves $5134 per patient due to lower agent costs and fracture morbidity costs, and prevents an average of 0.082 fractures per patient, resulting in a gain of 0.027 life years and 0.045 QALYs [88]. Therefore, the sequence of romosozumab to alendronate outperforms teriparatide to alendronate because it generates more QALYs at a lower cost.

The two studies above show that, while romosozumab has a higher unit cost, OP treatment is a lengthy process, and romosozumab can reduce the cost of subsequent treatment periods.

### Safety

Finally and most importantly, the safety of romosozumab is the most serious concern as a novel drug. The accompanying controversy has been constant since the development of the drug [89, 90]. As we mentioned before, sclerostin is a vital inhibitor Wnt/ β-catenin signaling pathway which plays a role in cardiovascular diseases and cancer [38, 91]. Therefore, the risk of carcinogenicity must be evaluated. A study in 2016 assessed the risk by a rat lifetime study. According to the findings of no effects on tumor incidence in rats, romosozumab would not pose a risk of human carcinogenicity [92]. Then, in the phase 3 FRAME study with the largest population, two serious cases of ONJ and AFF occurs within two years. Despite the small number of cases, there are enough to warrant attention. Because OP treatment is a lengthy process, the outcomes of prolonged treatment must still be confirmed. The author's response to the query was that confounding factors were present in both cases. One patient developed necrosis after receiving the first dosage of denosumab. Before the trial, one patient experienced pain at the atypical femoral fracture site [93]. There is no evidence of a patient with a lifelong sclerostin deficiency. Regarding the immune response problem, the author stated no differences in lymphocyte counts or infection rates in the FRAME study [93].

Simultaneously, cardiovascular AEs in the ARCH study have raised concerns about romosozumab safety. Concurrently, the post-marketing survey revealed that in the first romosozumab used in the clinic, 68 patients reporting serious cardiovascular AEs, with 16 of 68 patients died during March 2019 to September 2019 [94]. However, these were spontaneous reports with no control groups [95]. The evidence level was low. Nevertheless, European Medicines Agency (EMA) did not recommend using romosozumab in patients with myocardial infarction or stroke [96].

Because the Dickkopf-1 (DKK1) and sclerostin bind the LRP5/6 receptor complex and inhibit Wnt/β-catenin, expressed in vascular tissues and involved in vascular calcifications [97, 98]. Does a decrease in sclerostin cause a compensatory increase in DKK1 expression? Is it necessary to assess DKK1 levels to determine the cause of cardiovascular AEs? Is the risk for cardiovascular AEs by romozosumab affected by alendronate? Although the available studies did not demonstrate a definite link between romosozumab and cardiovascular events, there will be more debate in the future [99].

### Clinical efficacy

Romosozumab (EVENITY™), developed by Amgen and UCB, was first approved for marketing in Japan for treating OP in January 2019 [100]. This represents not only a success in developing the anti-sclerostin antibody, but also an important new option for doctors in treating OP. What about the clinical efficacy of romosozumab?

A pre-post study was conducted on 185 patients who received 6-month treatment with a 210 mg s.c. injection of romosozumab once every 4 weeks. No cases of new fractures were reported. There have been no reports of SAE associated with romosozumab. Compared with the baseline, the mean percentage BMD change were 6.34% (LS) and 1.53% (TH). sNTX were − 5.77% (at month 1), 1.88% (at month 3), and 1.74% (at month 6) from baseline. iP1NP were increased at month 1 (66.38%, p < 0.001), at month 3 (59.79%, p < 0.001) and at month 6 (38.61%, p = 0.001) [101].

Another 1-year romosozumab pre-post study was reported with 262 patients receiving romosozumab (210 mg s.c. Q4W) for 12 months. There were five new fractures reported, but no SAEs were reported. The mean percentage BMD change was 10.67% ± 0.8% (LS) and 2.04 ± 0.6% (TH), compared to the baseline at month 12. sNTX was − 3.70% (at month 1), 0.01% (at month 6), and 3.69% (at month 12) from baseline. iP1NP levels were higher at month 1 (77.34%), at month 6 (50.23%), and at month 12 (27.96%) [102].

The two pre-post studies demonstrated that romosozumab was safe and had a positive clinical effect. However, the follow-up period was short, and the sample size was small. More research is required in the future to comprehend romosozumab.

## Conclusion

There is no doubt that romosozumab is a groundbreaking drug in treating OP due to its novel character of inhibiting bone resorption while promoting bone formation. Romosozumab reaffirms the immense value of biomedical applications. For OP patients with a high fracture risk, romosozumab may be more beneficial than other OP medications. In some ways, Romosozumab can replace traditional medications for osteoporosis. However, romosozumab is a new drug that will not only be available until 2019, there is still much room for research in OP. Simultaneously, can romosozumab accelerate delayed healing or ununion, treat secondary OP safely and treat bone tumors? Will other emerging technologies like nanotechnology hold significant potential to impact the field of osteoporosis treatment in the future [103]? Perhaps, there will be answers in the future.

## Data Availability

Not applicable.

## Abbreviations

PM: Postmenopausal
OP: Osteoporosis
BMD: Bone marrow density
BTMs: Bone turnover markers
LRP5/6: Low-density lipoprotein-related receptors 5 and 6
AE: Adverse events
SAE: Serious adverse events
AFF: Atypical femoral fracture
ONJ: Jaw osteonecrosis
LS: Lumbar spine
TH: Total hip
FN: Femoral neck
FRAME: The Fracture Study in Postmenopausal Women with Osteoporosis
STRUCTURE: Open-Label Study to Evaluate the Effect of Treatment with Romosozumab or Teriparatide in Postmenopausal Women
ARCH: Active-Controlled Fracture Study in Postmenopausal Women with Osteoporosis at High Risk
BRIDGE: Placebo-Controlled Double-Blind Study Evaluating the Efficacy and Safety of Romosozumab in treating Men with Osteoporosis
s.c.: Subcutaneously
